# Gene-Environment Dependence Creates Spurious Gene-Environment Interaction

**DOI:** 10.1016/j.ajhg.2014.07.014

**Published:** 2014-09-04

**Authors:** Frank Dudbridge, Olivia Fletcher

**Affiliations:** 1 Department of Non-communicable Disease Epidemiology, London School of Hygiene and Tropical Medicine, London WC1E 7HT, UK; 2 Breakthrough Breast Cancer Research Centre, The Institute of Cancer Research, London SW7 3RP, UK; 3 Division of Breast Cancer Research, The Institute of Cancer Research, London SW7 3RP, UK

## Abstract

Gene-environment interactions have the potential to shed light on biological processes leading to disease and to improve the accuracy of epidemiological risk models. However, relatively few such interactions have yet been confirmed. In part this is because genetic markers such as tag SNPs are usually studied, rather than the causal variants themselves. Previous work has shown that this leads to substantial loss of power and increased sample size when gene and environment are independent. However, dependence between gene and environment can arise in several ways including mediation, pleiotropy, and confounding, and several examples of gene-environment interaction under gene-environment dependence have recently been published. Here we show that under gene-environment dependence, a statistical interaction can be present between a marker and environment even if there is no interaction between the causal variant and the environment. We give simple conditions under which there is no marker-environment interaction and note that they do not hold in general when there is gene-environment dependence. Furthermore, the gene-environment dependence applies to the causal variant and cannot be assessed from marker data. Gene-gene interactions are susceptible to the same problem if two causal variants are in linkage disequilibrium. In addition to existing concerns about mechanistic interpretations, we suggest further caution in reporting interactions for genetic markers.

## Main Text

There is much interest in discovering interactions between genetic and environmental risk factors for disease, because such interactions can shed light on biological processes leading to disease, identify subjects for whom risk factors are most relevant, and improve the accuracy of epidemiological risk models.^1^ Interaction is commonly understood as the modification by one factor of the effect of the other and is assessed statistically by testing for departure from additivity, on an appropriate scale, of the effects of gene and environment. Such a definition can be dependent on modeling assumptions and might not correspond to biological notions of interaction,^2–4^ but it is nevertheless useful in general exploratory settings.

To date, relatively few gene-environment interactions have been reported, in contrast with the large number of marginal associations discovered through genome-wide association studies (GWASs). One reason is that there might be relatively few subjects for whom the joint effect of gene and environment is high, so that very large samples are required to detect interactions. Another is that measurement error in either gene or environment can lead to substantial increases in required sample size.^5^ Thus, robust identification of a gene-environment interaction is regarded as a noteworthy finding.

In most studies, genotypes are measured not for the variants that directly affect disease but for markers in linkage disequilibrium (LD) with the causal variants. This is especially true in GWASs and other large-scale discovery studies that aim to map novel disease variants. This creates a misclassification problem in that the true causal variants have been measured with error. In contrast to common measurement error models, a marker is not an unbiased measurement of a causal variant (because the genotype frequencies of the marker and causal variant may differ), and the misclassification probabilities are unknown by design. General methods to adjust for measurement error^6^ are not applicable, and so we must accept possible bias in the estimation of interaction effects.

Through simulations, Hein et al.^7^ showed that the interaction effect of a marker is biased toward the null, with a corresponding increase in the sample size required for a study based on a marker. Garcia-Closas et al.^8^ showed analytically that measurement error in the environmental exposure also biases the interaction effect toward the null. Furthermore, Greenwood et al.^9^ showed that the interaction effect is not biased by measurement error in additional covariates included in the model. All of these studies assumed that the genetic marker and environmental exposure are independent in the source population or in the controls. Gene-environment independence also underlies the case-only design^10^ and extensions of it designed to improve the power of interaction tests.^11–14^ This assumption is often reasonable: for example, autosomal genotypes tend to be independent of sex. However, the properties of interaction tests have not been considered when gene and environment are not independent. Here we demonstrate that under gene-environment dependence, the interaction effect of a marker can be nonzero even if there is no interaction between the causal variant and the environment.

We and others recently established an association with breast cancer of the marker rs10235235, which maps to the *CYP3A* locus.^15^ This marker, which was initially identified through its association with urinary estrone glucuronide,^16^ a metabolite that is correlated with the sex hormone estradiol, is also associated with age at menarche. We found a statistical interaction between rs10235235 and age at menarche on breast cancer risk, which is therefore a gene-environment interaction under gene-environment dependence. However, rs10235235 is not known to be the causal variant, and we will show that the marker interaction does not imply an interaction at the causal variant.

Also in breast cancer, Nickels et al.^17^ established a statistical interaction between the marker rs3817198 at *LSP1* (MIM 153432) with parity, but also reported significant negative correlation between rs3817198 genotype and number of births. Again, this is a gene-environment interaction in the presence of gene-environment dependence, but rs3817198 is not known to be the causal variant.

As a further example, variants at chromosome 15q25 have been associated with both smoking and lung cancer.^18–20^ Interactions between these variants and smoking on lung cancer risk have also been identified,^19,21^ but not for the likely causal variants.^22^

Gene-environment dependence could arise in a number of ways. There is likely to be a genetic component to many of the established risk factors for which interactions are sought. In addition to the examples above, variants in genes involved in alcohol metabolism have been associated with alcohol intake,^23^ which is a risk factor for many diseases.^24^ GWASs have identified numerous variants associated with obesity, an established risk factor for many complex disorders including type 2 diabetes and breast cancer.^25^ Similarly, multiple variants that influence low-density lipoprotein cholesterol levels, one of the strongest risk factors for cardiovascular disease, have been identified.^26^ Even the more exogenous exposures, such as urban environment, might conceivably have a genetic component.^27^ However, on a per-gene basis, knowledge of biological function could be invoked to argue that a given gene is unlikely to affect an exposure of interest.

**Figure 1:**
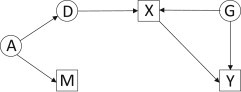
Directed Acyclic Graph Showing Gene-Environment Dependence by Mediation Boxes are observed variables, circles are unobserved, and arrows indicate directions of causal relationships. Abbreviations are as follows: D, causal variant; M, marker genotype; X, environmental exposure; Y, outcome such as disease; A, composite variable for ancestry, which gives rise to correlation (LD) between D and M; and G, composite variable for common causes of X and Y, which may include additional genes. X mediates the effect of D on Y. For example, a *CYP3A* variant (D) affects the risk of breast cancer (Y) via its effect on age at menarche (X).

**Figure 2:**
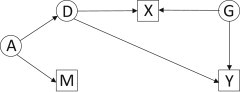
Directed Acyclic Graph Showing Gene-Environment Dependence by Pleiotropy Notation as in Figure 1. D has pleiotropic effects on X and Y, but there is no direct effect of X on Y. For example, a *CYP3A* variant (D) affects hormone levels, which independently affect age at menarche (X) and breast cancer (Y). Age at menarche is an independent risk factor for breast cancer because it marks additional causal processes (G).

**Figure 3:**
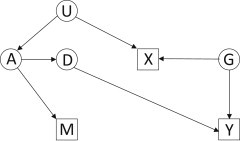
Directed Acyclic Graph Showing Gene-Environment Dependence by Confounding Notation as in Figure 1, with U an unmeasured confounder. D and X are associated by confounding. For example, haplotype frequencies at CYP3A (A) vary by latitude (U), as does age at menarche (X). In this graph, U is not a confounder of D and Y; such confounders are omitted for simplicity.

Figures 1, 2, and 3 illustrate three basic forms of gene-environment dependence. Association of a gene with an environmental risk factor is often taken to imply mediation of the genetic effect by the risk factor. That is, at least part of the effect of the gene on the outcome is via its effect on the environmental factor. For example, a variant at the *CYP3A* locus might directly affect levels of the hormone estradiol, which influences age at menarche, which then directly affects breast cancer risk (Figure 1).

On the other hand, the gene might have pleiotropic effects on the environmental factor and the outcome, but the environment might not cause the outcome. For example, a *CYP3A* variant might, via estradiol levels, influence both age at menarche and breast cancer risk. Age at menarche might have no direct effect on breast cancer, so it does not mediate the effect of the *CYP3A* variant, but it remains a risk factor by acting as a marker for other mechanisms that affect disease (Figure 2).

Gene-environment dependence could also arise through confounding, of which the principal source is population structure. For example, some *CYP3A* haplotypes might have become less frequent at northern latitudes. For unrelated reasons, age at menarche tends to be higher at northern latitudes, leading to an association with *CYP3A* variants (Figure 3). This confounding might be independent of any confounding of the gene and outcome and cannot be corrected using the standard methods to adjust for gene-outcome confounding.

Any combination of the above three forms might occur in practice, so for example a pleiotropic gene might affect the outcome through several pathways, only one of which is mediated by the environment of interest. The corresponding graph would be a combination of Figures 1 and 2, including both direct and indirect effects of the causal variant.

To formalize the interaction effects under gene-environment dependence, let *M* denote a genotyped marker, coded numerically, *X* an environmental exposure, and *Y* an outcome of interest. Consider a generalized linear model

$$E\left(Y|M,X\right)=h^{-1}\left(\beta_{0}+\beta_{M}M+\beta_{X}X+\beta_{MX}MX\right),$$

where *h* is a known link function. Writing *η*_*m*,*x*_ = *β*_0_ + *β*_*M*_*m* + *β*_*X*_*x* + *β*_*MX*_*mx*, the interaction term in this model is

(Equation 1) $$\beta_{MX}=\eta_{1,1}-\eta_{0,1}-\eta_{1,0}+\eta_{0,0}.$$

Let *D* denote the unmeasured genotype of the causal variant, with a corresponding generalized linear model in *D* and *X*

$$E\left(Y|D,X\right)=h^{-1}\left(\beta_{0}^{*}+\beta_{D}^{*}D+\beta_{X}^{*}X+\beta_{DX}^{*}DX\right),$$

where the asterisks denote effects in the model for *D* rather than for *M*. If the marker has no effect on the outcome, conditional on *D*, then

(Equation 2) $$\begin{array}{l} E\left(Y|M,X\right)=\sum_{d}E\left(Y|d,X\right)\mathrm{Pr}\left(d|M,X\right)\\ h^{-1}\eta_{m,x}=\sum_{d}h^{-1}\left(\beta_{0}^{*}+\beta_{D}^{*}d+\beta_{X}^{*}x+\beta_{DX}^{*}dx\right)\mathrm{Pr}\left(d|m,x\right) \end{array}.$$

The conditional distribution Pr(*D*|*M*,*X*) accounts for both the LD between marker and causal variant and the dependence between exposure and causal variant. Equations 1 and 2 allow the marker interaction term to be nonzero even when the interaction term for the causal variant is zero. Some conditions under which the marker interaction term is in fact zero are given in the following lemma.

**Lemma**
If $\beta_{DX}^{*}=0$, then *β*_*MX*_ = 0 if any of the following conditions hold
(1) there is no main effect of the causal variant on the outcome, $\beta_{D}^{*}=0$
(2) the marker is perfectly correlated with the causal variant, *D* = *M*
(3) the causal variant is independent of the marker, conditional on the exposure, Pr(*D*|*M*,*X*) = Pr(*D*|*X*)
Furthermore, under linear (*h*(*x*) = *x*) or log-linear (*h*(*x*) = log(*x*)) regression, *β*_*MX*_ = 0 if
(4) the causal variant is independent of the exposure, conditional on the marker, Pr(*D*|*M*,*X*) = Pr(*D*|*M*)
**Proof**
If $\beta_{DX}^{*}=0$ then the terms in *β*_*MX*_ are explicitly
$h^{-1}\left(\eta_{1,1}\right)=\sum_{d}h^{-1}\left(\beta_{0}^{*}+\beta_{D}^{*}d+\beta_{X}^{*}\right)\mathrm{Pr}\left(d|M=1,X=1\right)$
$h^{-1}\left(\eta_{1,0}\right)=\sum_{d}h^{-1}\left(\beta_{0}^{*}+\beta_{D}^{*}d\right)\mathrm{Pr}\left(d|M=1,X=0\right)$
$h^{-1}\left(\eta_{0,1}\right)=\sum_{d}h^{-1}\left(\beta_{0}^{*}+\beta_{D}^{*}d+\beta_{X}^{*}\right)\mathrm{Pr}\left(d|M=0,X=1\right)$
$h^{-1}\left(\eta_{0,0}\right)=\sum_{d}h^{-1}\left(\beta_{0}^{*}+\beta_{D}^{*}d\right)\mathrm{Pr}\left(d|M=0,X=0\right)$
If $\beta_{D}^{*}=0$ then
$h^{-1}\left(\eta_{1,1}\right)=\sum_{d}h^{-1}\left(\beta_{0}^{*}+\beta_{X}^{*}\right)\mathrm{Pr}\left(d|M=1,X=1\right)=h^{-1}\left(\beta_{0}^{*}+\beta_{X}^{*}\right)\eta_{1,1}=\beta_{0}^{*}+\beta_{X}^{*}$
Similarly, $\eta_{0,1}=\beta_{0}^{*}+\beta_{X}^{*}$ and $\eta_{1,0}=\eta_{0,0}=\beta_{0}^{*}$, so *β*_*MX*_ = 0 proving (1).
If marker and causal variant are perfectly correlated, then trivially $\beta_{MX}=\beta_{DX}^{*}=0$, which proves (2).
If Pr(*D*|*M*,*X*) = Pr(*D*|*X*), then *η*_1,1_ = *η*_0,1_ and *η*_1,0_ = *η*_0,0_, which proves (3).
Finally, if Pr(*D*|*M*,*X*) = Pr(*D*|*M*) and either *h*(*x*) = *x* or *h*(*x*) = log(*x*), then
$\begin{array}{l} \eta_{1,1}=\beta_{0}^{*}+\beta_{X}^{*}+\sum_{d}h^{-1}\left(\beta_{D}^{*}d\right)\mathrm{Pr}\left(d|M=1\right)\\ \eta_{1,0}=\beta_{0}^{*}+\sum_{d}h^{-1}\left(\beta_{D}^{*}d\right)\mathrm{Pr}\left(d|M=1\right)\\ \eta_{1,1}-\eta_{1,0}=\beta_{X}^{*} \end{array}$
Similarly, $\eta_{0,1}-\eta_{0,0}=\beta_{X}^{*}$ under either link function, so *β*_*MX*_ = 0 as required, which proves (4). Q.E.D.

Conditions (1)–(3) are reassuring because they mean that when a marker-exposure interaction exists, the marker must be associated with a causal variant. Furthermore, if the causal variant is independent of the exposure, then (4) shows that a marker-exposure interaction implies a causal variant-exposure interaction, at least under linear or log-linear regression (notably, this does not apply to logistic regression, although for rare outcomes *β*_*MX*_ will approach 0). However, if there is dependence between the causal variant and the exposure, then a marker-exposure interaction does not imply a causal variant-exposure interaction. Therefore, tests of marker-exposure interaction are not valid tests of interaction between causal variant and exposure.

We illustrate this with a numerical example. Consider a biallelic marker with population minor allele frequency (MAF) 0.1. The risk allele of the causal variant is present on half the chromosomes with the minor marker allele, but on no other chromosomes. So the MAF of the causal variant is 0.05, and the two loci are in linkage disequilibrium (D’ = 1, *r*^2^ = 0.47). If the risk allele has risk ratio 2, then assuming multiplicative risks and Hardy-Weinberg equilibrium, some simple calculations give the risk ratio for the marker as 1.5 (Table 1 and Appendix A).

Now consider a binary environmental exposure such that the risk ratio for the causal variant on the exposure is 1.5. No main effect of the exposure is assumed, although this does not matter in this example. Assuming that the quantities in Table 1 apply to unexposed subjects, some further calculations give the risk ratio for the marker as 1.6 in the exposed and 1.5 in the unexposed subjects (Table 2 and Appendix A). This reveals an interaction between the marker and the exposure on the risk of disease, although there is none for the causal variant. We regard this interaction as spurious, because it does not correspond to an interaction at the causal variant.

The spurious interaction arises from imperfect LD between the marker and causal variant, causing a misclassification error that differs between cases and controls, owing to the main effect of the causal variant, but that also differs between exposed and unexposed subjects, owing to the causal variant-exposure association. It is important to note that the spurious interaction cannot be removed by transformation of variables, as can be done in other cases,^4^ but is a direct result of measurement error of the causal variant. It does not depend on the mechanism of gene-environment dependence, of which Figures 1, 2, and 3 show a few examples, but arises from simple algebra of the statistical model.

A particular difficulty is that the bias depends on the causal variant-exposure association, which cannot be assessed from the marker data. Indeed, the marker might not show association with the exposure, even if the causal variant is associated with both. Even if the marker is associated with the exposure, it is unclear whether or not the causal variant would be independent of the exposure after conditioning on the marker, as required by the lemma. Therefore, any test of marker-environment interaction is potentially suspect because it cannot be determined from the data whether the causal variant is associated with the exposure, conditional on the marker.

Across the thousands of markers included in GWASs and targeted array studies, it is likely that some will be in LD with causal variants that are associated with the exposure of interest. The fact that few gene-environment interactions have been reported suggests that the magnitude of the bias is small. Indeed, under a typical scenario for current GWASs in which marker and causal variant have the same MAF of 0.2, their correlation is *r*^2^ = 0.8, and the causal variant has odds ratio 1.1 with both disease and a binary exposure, then similar calculations to those in Table 2 give the interaction odds ratio for the marker as 1.000433. More than a million cases and controls would be needed to detect this effect with 80% power at p < 0.05.^28^

However, higher interaction odds ratios can arise if the causal variant and marker have differing MAF. As an example, the marker rs10235235 observed at the *CYP3A* locus^15^ has MAF 0.09 in women of European ancestry and odds ratios for breast cancer of 0.979 (95% CI: 0.915–1.047) and 0.906 (95% CI: 0.864–0.950) in women with age at menarche ≤12 years and >12 years, respectively. This gives an interaction odds ratio of 1.08 (95% CI: 0.990–1.176) (the original study used a finer categorization of age at menarche, leading to a significant interaction). Assume that the marker and causal variant have the maximum correlation given their MAFs (i.e., D’ = 1). Treating the odds ratios as risk ratios, we can use the approach shown in Table 2 to solve for the causal risk ratios on disease and on exposure that lead to the observed marker risk ratios, given a fixed causal MAF. For causal MAF of 0.05, the observed marker effects can arise from causal risk ratio 0.831 on disease and 0.116 on exposure. This seems unlikely because such a strong effect (0.116 = 1 / 8.62) would probably be detected by a linkage study, but this region was not identified in the largest linkage scan for age at menarche.^29^ Similarly, a causal MAF of 0.01 implies a causal risk ratio of 0.155 (= 1 / 6.45) on disease and 0.208 on exposure, which again seems strong considering the lack of evidence of linkage to breast cancer in this region. However, a causal MAF of 0.02 implies causal risk ratios of 0.577 (= 1 / 1.73) on disease and 0.187 (= 1 / 5.36) on exposure, which is more plausible. Therefore, our observed marker interaction is compatible with a low-frequency causal variant with strong main effects but no interactions. Although common SNPs are generally expected to tag common causal variants,^30^ the possibility of a low-frequency causal variant suggests caution in claiming a gene-environment interaction in this case.

We have focused on gene-environment interaction, but gene-gene interaction is likewise of high interest and is also susceptible to this problem. There, a spurious interaction arises if two causal variants are in LD and at least one is measured with error, such as by a marker SNP. Recently, Hemani et al.^31^ have reported numerous *cis* interactions between marker SNPs on gene expression levels. However, many of the interactions can be explained by single variants in LD with both of the interacting markers (A.R. Wood, personal communication). In those cases the two causal variants are one and the same: of course a single variant is in LD, but cannot interact, with itself.

Measurement error can arise not only from a marker in LD, but also from the numerical coding of the genotype. If, for example, the true effect of a causal variant is dominant, but it is coded as additive and the linear model is otherwise correct, then the miscoding could also lead to a spurious interaction term. Furthermore, use of imputed rather than directly measured genotypes also creates measurement error, particularly for causal variants with strong effects because imputation is usually performed assuming no association with the outcome. Finally, exchanging the roles of gene and environment reveals that measurement error in the exposure could also create a spurious interaction even if the genotype is accurately measured.

The spurious interactions we have described are not a serious problem when the aim is to construct epidemiological models of risk, perhaps for disease prediction, in which case model fit may be improved by interaction terms. The real difficulty is with inference of biological interaction from statistical models, and our observations add to established concerns over the interpretation of statistical interactions that are model dependent.^3^ We believe that additional caution is required in the interpretation of gene-environment interactions, to allow for the possibilities of gene-environment dependence and imperfect LD between marker and causal variant. We suggest that sensitivity analyses such as that described above ought to be routinely performed to reduce the possibility of false positive reports of interaction.

## Figures and Tables

**Table 1:** Example Haplotype Frequencies for Disease and Marker Loci and Calculation of the Risk Ratio of the Marker

**Frequency**	**D = 0**	**D = 1**	**Total**	**Pr(Y = 1,D,M)**	**D = 0**	**D = 1**	**Total**	**RR(M)**
M = 0	0.9	0	0.9		0.9	0	0.9	1.0
M = 1	0.05	0.05	0.1		0.05	0.1	0.15	1.5

Abbreviations are as follows: D, allele at causal variant; M, allele at marker locus; Y, disease phenotype. Risk ratio (RR) of D = 1 is 2. Pr(Y = 1,D,M) is relative to a baseline that cancels in the marker risk ratio; see Appendix A for details of calculations.

**Table 2:** Example Haplotype Frequencies and Calculation of the Marker Risk Ratio in Unexposed and Exposed Subjects

**Pr(D,M|X = 0)**	**D = 0**	**D = 1**	**Total**	**Pr(Y = 1,D,M|X = 0)**	**D = 0**	**D = 1**	**Total**	**RR(M|X = 0)**
**Unexposed**								

M = 0	0.9	0	0.9		0.9	0	0.9	0.9/0.9 = 1
M = 1	0.05	0.05	0.1		0.05	0.1	0.15	0.15/0.1 = 1.5

**Exposed**								

M = 0	0.9	0	0.9		0.9	0	0.9	0.9/0.9 = 1
M = 1	0.05	0.075	0.125		0.05	0.15	0.2	0.2/0.125 = 1.6

Abbreviations are as follows: D, allele at causal variant; M, allele at marker locus. Risk ratio (RR) of D = 1 is 2 on disease Y and 1.5 on exposure X. Pr(Y = 1,D,M|X) are relative to baselines that cancel in the marker risk ratio; see Appendix A for details of calculations.

## Appendix A

### Marker Risk Ratio in Unexposed

The frequency of haplotype D,M in unexposed subjects is Pr(*D*,*M*|*X* = 0). The joint probability of an affected subject and haplotype D,M is

$$\mathrm{Pr}\left(Y=1,D,M|X=0\right)=\mathrm{Pr}\left(Y=1|D,M,X=0\right)\mathrm{Pr}\left(D,M|X=0\right)=R_{D}\mathrm{Pr}\left(Y=1|D=0,M,X=0\right)\mathrm{Pr}\left(D,M|X=0\right)$$

where R_D_ is the causal variant risk ratio. The risk of disease in an unexposed subject with marker M is

$$\mathrm{Pr}\left(Y=1|M,X=0\right)=\mathrm{Pr}\left(Y=1,M|X=0\right)/\mathrm{Pr}\left(M|X=0\right)=\sum_{d}\mathrm{Pr}\left(Y=1,D=d,M|X=0\right)/\sum_{d}\mathrm{Pr}\left(D=d,M|X=0\right).$$

The risk ratio for the marker is then

$$\frac{\mathrm{Pr}\left(Y=1|M=1,X=0\right)}{\mathrm{Pr}\left(Y=1|M=0,X=0\right)}=\frac{\sum_{d}R_{d}\mathrm{Pr}\left(d,M=1|X=0\right)\sum_{d}\mathrm{Pr}\left(d,M=0|X=0\right)}{\sum_{d}R_{d}\mathrm{Pr}\left(d,M=0|X=0\right)\sum_{d}\mathrm{Pr}\left(d,M=1|X=0\right)}.$$

### Marker Risk Ratio in Exposed

Note that

$$\mathrm{Pr}\left(Y=1,D,M|X=1\right)=\mathrm{Pr}\left(Y=1,X=1|D,M\right)\mathrm{Pr}\left(D,M\right)/\mathrm{Pr}\left(X=1\right)=S_{D}\mathrm{Pr}\left(Y=1,X=0|D,M\right)\mathrm{Pr}\left(D,M\right)/\mathrm{Pr}\left(X=1\right)=S_{D}\mathrm{Pr}\left(Y=1,D,M|X=0\right)\mathrm{Pr}\left(X=0\right)/\mathrm{Pr}\left(X=1\right)$$

where S_D_ is the risk ratio of D on the exposure X.

Also

$$\mathrm{Pr}\left(D,M|X=1\right)=\mathrm{Pr}\left(X=1|D,M\right)\mathrm{Pr}\left(D,M\right)/\mathrm{Pr}\left(X=1\right)=S_{D}\mathrm{Pr}\left(X=0|D,M\right)\mathrm{Pr}\left(D,M\right)/\mathrm{Pr}\left(X=1\right)=S_{D}\mathrm{Pr}\left(D,M|X=0\right)\mathrm{Pr}\left(X=0\right)/\mathrm{Pr}\left(X=1\right)$$

So the marker risk ratio in the exposed is

$$\frac{\mathrm{Pr}\left(Y=1|M=1,X=1\right)}{\mathrm{Pr}\left(Y=1|M=0,X=1\right)}=\frac{\sum_{d}S_{d}R_{d}\mathrm{Pr}\left(d,M=1|X=0\right)\sum_{d}S_{d}\mathrm{Pr}\left(d,M=0|X=0\right)}{\sum_{d}S_{d}R_{d}\mathrm{Pr}\left(d,M=0|X=0\right)\sum_{d}S_{d}\mathrm{Pr}\left(d,M=1|X=0\right)}$$

The above calculations may be performed using Table S1 available online.

## Supplemental Data

Table S1. Calculations of Interaction Risk Ratio Instructions for use are included in the spreadsheet.
